# Mental health responses in countries hosting refugees from Ukraine

**DOI:** 10.1192/bjo.2022.55

**Published:** 2022-04-01

**Authors:** Kenneth R. Kaufman, Kamaldeep Bhui, Cornelius Katona

**Affiliations:** Departments of Psychiatry, Neurology and Anaesthesiology, Rutgers Robert Wood Johnson Medical School, New Brunswick, New Jersey, USA; and Department of Psychological Medicine, Institute of Psychiatry, Psychology & Neuroscience, King's College London, UK; Department of Psychiatry and Nuffield Department of Primary Care Health Sciences, University of Oxford, UK; East London NHS Foundation Trust, UK; Oxford Health NHS Foundation Trust, UK; and World Psychiatric Association Collaborating Centre in Research, Training, Policy and Practice, Oxford, UK; Helen Bamber Foundation, London, UK; and Division of Psychiatry, University College London, UK

**Keywords:** Refugee, asylum seeker, migrant, trauma and mental health, humanitarian and moral obligations, safety, social support, interventions, cultural competence, Ukraine refugee crisis, global response, adverse childhood experiences, healthcare policy and policy makers

## Abstract

The Ukrainian refugee crisis highlights the many issues associated with trauma, distress, mental and physical health, culturally competent assessments, and meaningful support and interventions. This crisis requires international support and a global response, as hosting countries have specific competencies and capacities. The authors hope that the groundswell of international concern over the crisis in Ukraine will lead not only to a comprehensive response to the needs of refugees from that country but also to a recognition of the needs of other asylum seekers and refugees and to our collective moral obligation to address those needs equitably.

At the time of writing, more than 10 million people from Ukraine are internally or externally displaced, of whom more than 3.8 million have left Ukraine seeking refuge elsewhere in Europe.^1,2^ It is estimated that the total number of externally displaced people from Ukraine will rise to about 4 million.^3^ This clearly represents a huge humanitarian crisis, although it must also be remembered that there are currently more than 80 million displaced persons worldwide, of whom more than 26 million are refugees and more than 4 million are asylum seekers.^4^ Most people seeking refuge do so first in countries neighbouring their countries of origin. There are already over 2.2 million displaced people from Ukraine in Poland.^1^ Some have travelled through multiple countries to be close to family and friends.

A breakdown in international political relationships and a unilateral initiation of war by Russia triggered the most recent crisis in Ukraine. The Russian press argue that their country is not attacking civilians but fighting discrimination against Russians in Ukraine and trying to keep the peace and demilitarise Ukraine as well as combat what they term as ‘Nazism’.^5^ A battle of dominant narratives and the promotion of myths are familiar accompaniments to war. The killing of innocents and alleged war crimes are deeply disturbing experiences, for those close to the theatre of war and for observers, as deeply held fundamental truths and moral assumptions are challenged. At times of war the truth matters more than ever.

The solution to such a crisis must be political, with lessons learned from the many all too similar displacement crises that have littered modern history. In this context it is important to remember the extraordinary humanitarian response by the German government to the Syrian refugee crisis in 2015. This involved not only opening the borders but also dramatically increasing active resettlement, relocation and offers of humanitarian admission.^6^ The fundamental principle was that refugees should be welcomed rather than merely allowed and that they should be supported to become citizens, to contribute their talents in their new homes and to remain able to return to their country of origin should they wish to once the crisis was over – which, of course, is still not the case in Syria. Where the conflict is persistent, people do make homes in host countries, and the evidence is that they make substantial contributions financially, economically and culturally to enrich the receiving nations.^7^

## The mental health and psychosocial impact of displacement

A comprehensive response to the Ukraine crisis must address the mental health and psychosocial challenges faced by refugees as well as by those who are internally displaced. The UN has two ongoing plans.^8,9^ It is now the norm for humanitarian responses to address both psychosocial and mental health needs.^10^ A systematic review of prevalence studies (all based on clinical interviews and validated diagnostic systems) reported that, overall, 31.5% of refugees had post-traumatic stress disorder (PTSD), 31.5% had depressive disorders, 11.1% had anxiety disorders and 1.5% had psychoses.^11^ The rates for PTSD and depression are dramatically higher than those in the general population.

These are, of course, not the only disorders found within a refugee population. Many will have ‘complex’ PTSD as result of the multiple and repeated trauma they have experienced. Issues of trust, loss of ‘agency’, inability to imagine a personal future, inappropriate risk-taking and somatisation are common presenting features of such complex PTSD.^12^ Those not displaying signs and symptoms will be carrying a greater allostatic load and are likely to suffer poor physical and mental health in the longer term.^13^ A significant proportion will have alcohol and substance use disorders which may be precipitated or aggravated by trauma. Others will have intellectual disabilities, which may not be identified and which may increase their difficulties in coping both with trauma and with a new environment. Comorbid mental disorders are common in refugees. For example, complicated grief reactions accompany loss events (life, possessions and place) when social supports and other community assets are destroyed or removed. Furthermore, comorbid depression and post-traumatic symptoms and anxiety states are common. People with pre-existing severe mental illnesses (psychosis, affective states, intellectual disability and neuropsychiatric disorders) are vulnerable to further relapses related to the direct traumatic impacts of war, the disruption of care services, and potential isolation, abandonment and homelessness. There again they may present with combinations of post-traumatic stress, affective symptoms, emotional dysregulation and fluctuating psychotic symptoms. There is a need to build a stronger evidence base with better quality studies investigating the prevalence and social, contextual and political drivers of poor mental health among refugees and asylum seekers.^14^

There are many factors contributing to the high prevalence rates of significant mental illness. Pre-migration factors include the experiencing and witnessing of war violence and other atrocities, including rape and other sexual violence. Many refugees will have experienced loss and trauma on their journeys, which may include human trafficking and other forms of exploitation. Furthermore, loss of the opportunity to follow religious practices and other protective factors may have a negative impact on the mental well-being of many displaced persons – both internally displaced and refugees.

## Overall humanitarian support: a global moral imperative

Refugees are also vulnerable to multiple post-migration factors increasing their vulnerability to mental health problems. These include separation from and uncertainty about family and friends, loss of previous support networks, difficulty in accessing health and social care, and the challenges of securing stable work and accommodation. A national strategy to minimise these challenges is a key component of a truly welcoming host country's political response to the crisis. In this context, the international community has a moral duty to ensure that countries neighbouring a crisis zone (such as Lebanon and Jordan for Syria, Pakistan and Iran for Afghanistan and Poland for Ukraine) are not required to provide a level of support beyond their capacity to do so. There may be different levels of receptivity and capacity to accommodate so many refugees, especially when occurring in such a brief period of time, and to provide them with sufficient support, schooling and healthcare. Yet, the Ukraine crisis is a global emergency. In such situations, humanitarian principles must override other considerations, and governments and the international community have a moral duty to ensure that the necessary resources are provided, just as happened with the COVID-19 pandemic.

Such overall support is vital and perhaps more important than offering specific mental health interventions or uncritically accepting that mental disorders are common and a priority for intervention. Indeed, the notion of PTSD being an inevitable consequence of natural disasters and conflict has been challenged by many as representing the medicalisation of suffering and an overly dispassionate and unhelpful response. The real need is for practical support, including jobs, welfare, housing, safety and education for children. However, the provision of such services can lead to hostility in the receiving country, from ordinary people fearing for their jobs to holding on to and even hardening prejudicial views about refugees. Such hostility (and consequent discrimination and potential for actual violence), together with poverty, multiple adversities and isolation from friends and family, add to the burden refugees face, not only from traumatic journeys and conflict in their home countries, but also from lack of employment opportunities and the lengthy and uncertain asylum processes they face in the countries offering asylum.

## Immigration policies, public health strategies and other potential barriers

Furthermore, immigration is a contentious topic of political discourse, with political parties in any country often having strong opposing views. Many governments create legal and policy barriers to immigration in general, which reflects a hostility that (consciously or inadvertently) can shape the response to refugees. The conditions in the UK, for example, currently include a review of immigration legislation^15^ which (in the opinion of the UNHCR) will substantially increase the barriers asylum seekers and refugees face in the process of becoming British citizens.^16^ Britain's colonial past is littered with examples of differential handling of asylum seekers and migrants from the former colonies and other countries, especially for people with origins in Africa, South Asia or the Caribbean. In this context, it is difficult to escape the conclusion that hostile legislation is a manifestation of structural racism.^17^

A public health strategy, and more specifically a public mental health strategy, is central to an effective response. Such a strategy must recognise both the systemic and the intrinsic barriers to accessing the mental healthcare that many refugees will need, as well as potential ‘enablers’ such as positive policies and active social support.^18^

Systemic barriers include lack of knowledge, understanding and training within primary and specialist mental health teams regarding refugees’ complex health needs and their cultural diversity. Scarcity of skilled interpreting services, lack of sustained funding for and joined-up thinking across the public and the voluntary sector, and lack of time in an overstretched public health service must all be addressed.

Intrinsic barriers to accessing mental healthcare include refugees’ lack of familiarity with healthcare services and entitlements, conflicting models of health and illness, fear of the effects of disclosing mental health vulnerability, stigma, shame and self-blame and the effects of trauma on autonomy and on the ability to trust those attempting to help and support them.

## Comprehensive health assessment programmes to welcome refugees

Assessment of the possible presence or emergence of significant mental illness is a key component of the initial welcoming process for individual refugees and refugee families. Such assessment should be trauma-informed.^19^ A trauma-informed approach includes acknowledgement of the links between trauma and mental health and of the broad range of mental responses to trauma. It prioritises trustworthiness and transparency and emphasises a reflective, gentle and collaborative approach to assessment, care and support. It avoids overemphasising the distinction between caregiver and care recipient, requiring that strict criteria are met, and assessment of those criteria through a dispassionate and interrogative process that treats those seeking asylum as potential security threats or criminals. Basic security checks are entirely compatible with a rapid, responsive and timely process to consider asylum cases, whereas lengthy asylum processes add to the burden of mental illness and should be avoided.^20,21^ Recognising and addressing the potential for vicarious trauma in caregivers is also crucial.

Many refugees will not find it easy to recover from loss of family and friends and social networks. Moral frameworks are established from childhood through adulthood and reflect societal expression of truth, justice, liberty and conditions that permit people to flourish. Moral frameworks require a basic trust in goodness and in society as a positive force, rather than a destructive one that shatters their worldviews and becomes authoritarian and terrorising rather than respecting citizenship and humanitarian and legal rights.

Refugees often also have poor physical health with complex comorbidities, including a high risk of personal injuries, exposure to communicable disease and undiagnosed or poorly treated chronic medical conditions. There is therefore a need for a comprehensive screening health assessment incorporating physical, mental and social health concerns.^22^ This should include screening questions to identify trafficking/modern slavery, sexual exploitation, female genital mutilation (FGM) and previous torture.

Any comprehensive mental health assessment of newly arrived refugees must be culturally competent with an appreciation of the variety in cultural expression of mental illness, symptoms and stigma in the originating and the host countries.^23^ The range of necessary competencies has been articulated in an article in the *BJPsych Open* Refugee and Asylum Mental Health Thematic Series.^24^ Clinicians, systems leaders and policy makers will need multiple competencies in the following areas:

- narrative methods and cultural psychiatric critiques and assessment methods
- assessment of symptoms out of cultural context
- trauma studies and social and neuroscience perspectives on recovery and therapeutics
- migration studies, legal and social perspectives on protecting health and recovery
- liaison, medical and public health actions
- cultural adaptation of existing therapeutics and outcome measures and policy.

The social determinants of mental and physical illness should also be assessed in a structured manner, for example using the American Academy of Family Physicians’ checklist^25^ and the American Heart Foundation's guidance.^26^

While recognising the high prevalence rates of significant mental illness it is also important not to overmedicalise distress and associated adaptive and transient emotional symptoms and to consider the trajectory of distress over time.^27^ Key questions for the assessor include establishing whether there are multiple mental symptoms, whether they are persistent, the extent to which they are disabling and whether they exceed what would be regarded as culturally appropriate. The UNHCR has proposed an umbrella classification of ‘emotional disorders’ which covers depression, anxiety and PTSD and is characterised by the presence of overwhelming sadness/apathy, highly distressing re-experiencing symptoms and/or exaggerated or uncontrollable anxiety or fear persisting for at least 2 weeks.^28^

Documentation of torture, racism, discrimination and violence are often helpful and necessary in making a case for residency rights, as is the presence of a mental illness, yet the assessment necessary for this may encourage overmedicalisation and a focus on providing specific mental health interventions alone rather than on overall needs such as housing and support.

## The particular needs of refugee children

It is vital to prioritise and give full consideration to the needs of refugee children – both those arriving unaccompanied and those within refugee families. Abuse and torture can have profound psychological, developmental and physical impacts on children. Adverse childhood experiences include physical and sexual abuse (trafficking being a particular risk for unaccompanied minors), discrimination and racism, food poverty (going to bed hungry), parents who are incarcerated (all too often part of the process in asylum applications) and parental mental illness, including drug use. Abuse, neglect, severe stress and adverse events in childhood all increase the long-term risk of physical, mental, emotional, social and relational health problems. Indeed, children facing six or more adverse experiences have a life expectancy reduced by up to 20 years.^29^ Children being evacuated, sleeping in shelters, seeing fear and death around them, and travelling long distances to escape war and hostility, are likely to suffer adverse effects as a result of these experiences. They will need safety first, then support, social care and therapeutic interventions. Early investment in prevention and in timely intervention will protect children's health and well-being and promote their sustained recovery from trauma.

## Cross-sector partnerships for effective care pathways

Treatment of mental health problems in refugees must be integrated into an overall care plan which ensures that basic needs are met, that essential services can be accessed and that refugees are protected from further harm. Connecting with culturally, linguistically and therapeutically appropriate and accessible services in the voluntary, charity and commissioned sectors is essential. Partnerships between these agencies are an essential prerequisite of an effective response. This in turn requires a ‘pyramid of care’ approach^30^ with locally determined and properly articulated care pathways. Case management can help identify needs, strengths and resources and ensure interdisciplinary team coordination. Brief psychotherapies can be ‘manualised’ and delivered by non-specialists following brief training and with appropriate clinical supervision, with specialist referral and care made available for more serious and persisting mental symptoms. Refugees who have experienced multiple and/or repeated trauma often require more individually tailored psychotherapy focusing initially on stabilisation through the understanding and control of symptoms, followed by work on processing of traumatic memories and a final phase of social and psychological integration.

## Hope for change

Is it too much to hope that the groundswell of international concern over the crisis in Ukraine will lead not only to a comprehensive response to the needs of that country's refugees but also to a recognition of the needs of other asylum seekers and refugees and of our collective moral obligation to address those needs equitably?

## Data Availability

Data availability is not applicable to this editorial as no new data were created or analysed in its preparation.
